# Peptide MegaPools Approach to Evaluate the Dengue-Specific CD4 and CD8 T-Cell Response

**DOI:** 10.3390/pathogens15010005

**Published:** 2025-12-20

**Authors:** Marta Tiberi, Linda Petrone, Andrea Salmi, Valentina Vanini, Gilda Cuzzi, Alessandra D’Abramo, Patrizia De Marco, Alba Grifoni, Daniela Weiskopf, Alessandro Sette, Emanuele Nicastri, Delia Goletti

**Affiliations:** 1 Translational Research Unit, National Institute for Infectious Diseases “L. Spallanzani”-IRCCS, 00149 Rome, Italy; 2 Professioni Sanitarie Tecniche, National Institute for Infectious Diseases “L. Spallanzani”-IRCCS, 00149 Rome, Italy; 3 Clinical Disease Unit, National Institute for Infectious Diseases “L. Spallanzani”-IRCCS, 00149 Rome, Italy; 4 Center for Vaccine Innovation, La Jolla Institute for Immunology (LJI), La Jolla, CA 92037, USA; 5 Division of Infectious Diseases and Global Public Health, Department of Medicine, University of California, San Diego (UCSD), La Jolla, CA 92093, USA

**Keywords:** dengue, dengue disease, dengue vaccination, T cell response, cross-reactive memory response, QDENGA vaccine, CD4 and CD8 T cell response

## Abstract

Background: Being central players in the adaptive immunity, the study of T-cell responses is crucial in both natural infections and vaccine-induced immunity. In this study, we assessed the antigen-specific T-cell responses to dengue virus (DENV) to identify the most immunogenic antigen for evaluating dengue-specific T-cell responses. Methods: Patients with dengue disease and subjects vaccinated with the QDENGA (TAK-003) vaccine (before and three months after vaccination) were enrolled. The T-cell-specific response was measured by ELISPOT and Activation Induced Markers (AIM) assay following PBMC stimulation either with DENV1-4 CD4 and CD8 MegaPools (MP) or serotype-specific DENV peptide pools at different concentrations. Results: We found that both DENV1-4 CD4 MP (at 1 µg/mL) and CD8 MP (at 5 µg/mL), which encompass all four DENV serotypes, elicited specific T-cell responses in patients with dengue infection independent of the infecting serotype. In contrast, selected serotype-specific DENV peptide pools have a lower ability to induce a measurable T-cell response. Moreover, DENV1-4 CD4 and CD8 MPs, at the highest concentrations, are suitable candidates to evaluate the dengue-specific T-cell response in vaccinated subjects. Conclusions: These findings support the use of the MP approach to investigate dengue-specific T-cell response to monitor the response during the infection and after vaccine administration.

## 1. Introduction

Dengue is an arboviral disease endemic in more than 100 countries across the tropical and subtropical regions of Asia, Africa, the Western Pacific, and the Americas [1]. In recent years, cases have also been reported in Europe and the United States [2]. Dengue virus (DENV) is primarily transmitted by mosquitoes, mainly *Aedes aegypti*, and less commonly by *Aedes albopictus*.

There are four distinct serotypes of the virus: DENV1, DENV2, DENV3, and DENV4 [3]. Each serotype consists of a single molecule of RNA approximately 11 kb in length. This genome encodes three structural proteins—Envelope (E), pre-membrane (prM), and Capsid (C)—and seven non-structural (NS) proteins: NS1, NS2a, NS2b, NS3, NS4a, NS4b, and NS5. Although ~65% of the amino acid sequence is conserved among dengue serotypes, notable variations have been identified in the E protein and NS1 [4,5].

Any of the four DENV serotypes can cause dengue disease. Most people with a primary dengue infection have mild or unnoticed symptoms that are rarely identified as dengue. In contrast, secondary infections may result in more severe and potentially life-threatening pathological conditions [6,7]. Specifically, earlier hypotheses suggested that antigen-specific T-cells generated by primary DENV infection are reactivated during a secondary infection. In cases of heterologous serotype, these T-cells, with low affinity for the epitopes of the secondary infecting serotype, can contribute to the development of severe dengue disease, a phenomenon known as “original antigenic sin” [8,9]. However, accumulating evidence shows that T-cells—including both CD4^+^ and CD8^+^ subsets—may play a protective role in dengue even modulating the severity of the disease [10,11,12,13]. The increased risk of severe dengue during a secondary infection is now widely accepted to be mediated by the antibody-dependent enhancement (ADE) phenomenon [14,15,16]. Indeed, the high genetic and antigenic similarity among DENV serotypes often results in cross-reactivity rather than effective protection [17]. In some instances, this cross-reactivity contributes to initiating the ADE process, when immune complexes of DENV and the IgG antibody bind to Fcγ receptors on antigen-presenting cells, facilitating viral entry, enhancing replication, and suppressing immune response [18]. These events in turn contribute to increased viremia and worsened clinical manifestations [10]. Thus, the progression to severe disease should be mostly associated with an imbalance among immune cell subsets (CD4^+^ T-cells, CD8^+^ T-cells, B cells, and innate immune cells) rather than a result of deficiency of only one subset or the overstimulation of another [11,19].

Previous research has identified potential differences in immunodominant epitopes after dengue infection. Indeed, primary infection with DENV3 induces immune responses against all 10 DENV proteins. However, DENV2 is more likely to induce responses targeting non-structural proteins [12,20]. Computational analysis among pan-DENV sequences revealed that at least 80% of sequences were conserved among four serotypes, and the majority are considered immune-relevant as T-cell determinants [21]. Thus, protein immunodominance is complex and largely focused on several protein targets, suggesting that both structural (C, prM, E) and non-structural (NS1-5) proteins are required to trigger an effective DENV-specific T-cell response [22,23]. Therefore, to assess the complexity of DENV-specific T-cell immunity following infection, peptides representing both structural and non-structural proteins are required. Commercially available single serotype-specific peptide pools provide partial coverage of each DENV serotype’s proteome [24]. Despite this limitation, peptide-based methods may allow for a detailed analysis of serotype-specific and cross-reactive T-cell responses in dengue patients [25]. However, testing large pools with hundreds of peptides presents technical difficulties due to solvent-induced toxicity. The MegaPool (MP) approach, developed using sequential lyophilization for large numbers of peptides, enables their use in various assays for measuring T-cell responses [such as enzyme-linked Immunospot (ELISPOT), intracellular cytokine staining (ICS), and activation-induced marker (AIM) assays] [26]. This method has been validated in various contexts, including infectious diseases, allergies, and autoimmunity [27]. Previous studies have developed DENV-specific MPs for CD4 and CD8 T-cells consisting of 180 and 268 peptides, respectively [28,29]. These pools are based on experimentally derived T-cell epitopes following infection from all four DENV serotypes. They were identified using samples derived from different geographical locations, thus allowing for a comprehensive assessment of DENV-specific responses after infection [11,28,29,30].

Vaccines are widely used for the prevention of infectious diseases. At present, five types of dengue vaccines exist, each with specific limitations [30]. An effective vaccine should offer protection against all four serotypes to help prevent transmission. QDENGA (TAK-003), a recombinant live-attenuated tetravalent vaccine, was recently developed [31]. This vaccine uses an attenuated DENV2 backbone and incorporates the premembrane (prM) and Envelope (E) genes from DENV1, DENV3, and DENV4 to target broad protection against dengue [32].

Therefore, the objective of this study is to identify the most effective peptide pools and to establish the optimal experimental conditions for evaluating dengue-specific T-cell responses instrumental for assessing dengue-specific T-cell responses following natural dengue infection or vaccination.

## 2. Materials and Methods

### 2.1. Study Population

Patients admitted to the National Institute for Infectious Diseases (INMI) L. Spallanzani-IRCCS with dengue disease were enrolled. Dengue infection was diagnosed by NS1 rapid test (STANDARD F Dengue NS1 Ag SD Biosensor, Suwon, Republic of Korea) at admission and confirmed by real-time polymerase chain reaction (RT-PCR). Subjects who were referred to the outpatient clinic at INMI and vaccinated with TAK-003 (Qdenga^®^), a live attenuated vaccine produced by Takeda (Tokyo, Japan), were enrolled as healthy donors before administration of the vaccine and three months after the first vaccine dose as vaccinated subjects. Exclusion criteria for all the enrolled subjects were HIV infection, autoimmune diseases, diabetes, or prior vaccination against Yellow Fever. Candidates to vaccines unable to provide consent for participation and with significant contraindications (e.g., a high risk of intolerance to the vaccine components) were excluded. Ethical Committee Lazio Area 4 (protocol number 96-2023/21 February 2024; protocol number 56-2023/4 December 2023) approved the study. Written informed consent was required to participate in the study.

### 2.2. Peripheral Blood Mononuclear Cells Isolation

Human heparinized blood samples were subjected to density gradient centrifugation using Ficoll-Paque Premium (GE Healthcare Biosciences, Chicago, IL, USA). The isolated peripheral blood mononuclear cells (PBMCs) were resuspended in fetal bovine serum (FBS) (Gibco Life Technologies, Carlsbad, CA, USA) containing 10% dimethyl sulfoxide (DMSO) and cryopreserved in liquid nitrogen until use.

### 2.3. Selection of Dengue Peptides

DENV1-4 CD4 MegaPool (MP) and DENV1-4 CD8 MP are synthetic peptide pools developed based on the identification of recognized epitopes. These two DENV-specific MPs were designed to combine a large number of peptides belonging to all four dengue virus serotypes into a single pool through sequential lyophilization. The DENV1-4 CD4 and CD8 MPs used in this study consist of 180 and 268 peptides and include 15-mers and 9/10-mers, respectively [12,28,29,33,34,35]. These peptides were pooled, lyophilized, reconstituted in DMSO, aliquoted, and stored at −20 °C until use. Peptide pools corresponding to the four single serotypes were obtained from BEI Resources (https://www.beiresources.org/ accessed on 27 October 2024). Each single pool contained the overlapping peptide pools (peptides are 12-mers to 20-mers) that spanned most of the dengue serotype genomes corresponding to DENV serotype 1 Singapore/S275/1990 (3 peptide pools—NR50710-NR2752-NR4203), DENV serotype 2 New Guinea C (8 peptide pools—NR505-NR506-NR508-NR2747-NR2748-NR509-NR2750-NR50709) [36,37], DENV serotype 3 Philippines/H87/1956 (1 peptide pool—NR9228), and DENV serotype 4 Singapore/S275/1990 (4 peptide pools—NR9229-NR2755-NR2756-NR4205) [37]. All the reagents were obtained through the NIH Biodefense and Emerging Infections Research Resources Repository, NIAID, NIH: Peptide Array, Dengue Virus. Single peptides were provided in lyophilized form and were reconstituted according to the manufacturer’s instructions using either DMSO or water. Peptides corresponding to each serotype were then pooled to generate a single peptide pool for each DENV serotype, aliquoted, and stored at −20 °C until use.

### 2.4. In Vitro Cell Stimulation and IFN-γ-ELISPOT Procedure

Thawed PBMCs were plated into flat-bottom plates (Mabtech (Nacka Strand, Sweden) Pro Human IFN-γ, ALP, 3420-2AST-2) and stimulated for 16–20 h with DENV1-4 CD4 MP, DENV1-4 CD8 MP, DENV1 pool, DENV2 pool, DENV3 pool, DENV4 pool at 0.1 µg/mL, 1 µg/mL, 5 µg/mL, Staphylococcal enterotoxin B (SEB, Sigma Aldrich, St. Louis, MO, USA) at 200 ng/mL as a positive control, and SARS-CoV-2 Spike-peptide pool (Miltenyi (Bergisch Gladbach, Germany) PepTivator^®^SARS-CoV-2 Prot_S1, Prot S, and Prot S^+^) at 0.1 µg/mL as an unrelated antigen [38]. Unstimulated PBMCs were included as a negative control. At least 50,000 cells were stimulated with SEB, and at least 150,000 cells were stimulated with or without the other stimuli. The IFN-γ-ELISPOT assay was performed according to the manufacturer’s instructions. Briefly, the cell suspension and stimuli were dispensed into each well of the plate and incubated at 37 °C for 16–20 h. Then, the cell suspension was discarded, and wells were washed five times with 200 µL of filtered phosphate-buffered saline (PBS). A 1:200 dilution of the Conjugate Reagent in PBS + 0.5% FBS was then added (100 µL per well), and the plate was incubated for 2 h at room temperature. After a second wash step, 100 µL of substrate was added to each well and incubated for 10–30 min, until spot formation. The plate was left to dry 24 h before analysis. Responses were considered positive based on two criteria: (1) the presence of more than 5 spot-forming cells (SFCs) per well in stimulated wells after subtracting the negative control, and (2) a stimulation index greater than 2 [39], defined as the number of SFC in the stimulated wells divided by the media of SFCs in the double unstimulated wells. Inter-assay variability was evaluated by standardizing the instrument’s reading parameters. For randomly selected experiments, readings were compared across the instruments and 2 different operators in a blinded manner.

### 2.5. Activation-Induced Markers (AIM) Assay by Flow Cytometry Analysis

Thawed PBMCs were cultured at a concentration of 1 × 10^6^/mL in 48 well plates and stimulated for 48 h [40,41] at 37 °C, 5%CO_2_ with DENV1-4 CD4 MP 5 µg/mL, DENV1-4 CD8 MP 5 µg/mL, SEB at 200 ng/mL as a positive control, and SARS-CoV-2 Spike-peptide pool at 0.1 µg/mL as unrelated antigen. AIM assay was performed using the following conjugated antibodies: anti-CD3 FITC, anti-CD137 APC (from Biolegend, San Diego, CA, USA), anti-CD4 PE, anti-CD8 BV786, anti-CD69 PE-CF594, anti-CD25 BV480, and anti-OX40 (CD134) BV421 (from BD Bioscience, San Jose, CA, USA). The viability stain A700 was also included. At least 100,000 lymphocytes were acquired using LxFLEX (Beckman Coulter, Brea, CA, USA). Multiple-parameter flow cytometry results were analyzed using FlowJo software (version 10.10.0, BD Life Sciences, Franklin Lakes, NJ, USA).

An antigen-specific response was considered positive if the proportion of stimulated cells, defined as CD25^+^CD134^+^ for CD4^+^ and CD137^+^CD69^+^ for CD8^+^, was at least two times greater than that of the unstimulated control, and if the number of gated events was at least 10 cells.

### 2.6. Statistical Analysis

All statistical analyses were performed using GraphPad Prism 8. The following tests were used: Wilcoxon test for paired data and Mann–Whitney U test for unpaired data and Chi-square test for categorical variables; receiver-operator characteristic analysis (ROC) and analysis of the Area Under Curve (AUC) were applied to define cut-off values for scoring purposes. *p*-values < 0.05, or resulting from Dunn’s correction for multiple comparisons, were considered statistically significant.

## 3. Results

### 3.1. Study Population Characteristics

We studied eight dengue patients infected with DENV1 (25%), DENV2 (37.5%), or DENV3 (37.5%), and six individuals before and after their first dengue vaccine dose. Demographics are presented in Table 1.

Among the DENV patients, six (75%) were Italian (Caucasian) and two (25%) were from Asia, with a female prevalence (62.5%). The median age was 42 years (IQR: 25–71). All DENV patients had non-severe dengue. At the time of enrollment, one out of eight individuals (12.5%) was experiencing a second DENV infection. This patient had a DENV2 infection at enrollment. The median interval between symptom onset and enrollment was 7 days (IQR: 4–9 days). The healthy donors/vaccinated subjects were all from Italy (100%) and equally distributed by sex (female 50%). The median age was 48 (25–65), with no statistically significant differences compared to dengue patients (*p* = 0.64).

### 3.2. Unlike Single DENV Serotype Pools, DENV1–4 CD4 and CD8 MPs Consistently Induce Dengue-Specific T-Cell Responses in Patients Infected with Different DENV Serotypes

To evaluate the dengue-specific T-cell response, we tested six peptide pools in patients diagnosed with dengue disease to determine which pool elicited the highest IFN-γ production.

All patients responded to SEB stimulation, confirming overall T-cell functionality (Figure S1); however, only 62.5% exhibited a recall response to the SARS-CoV-2 Spike peptide pool (Figure S1), possibly reflecting differences in various exposures or infections that dampened the immune response to the recall antigen Spike while enhancing the response to DENV.

**Figure 1:**
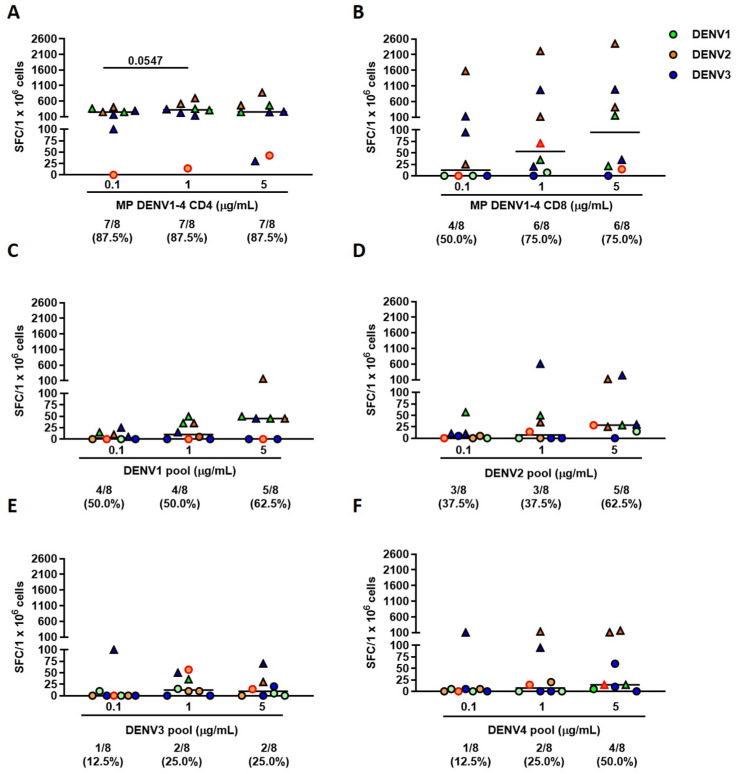
DENV1-4 peptide MegaPools (MPs) induce IFN-γ-producing T-cells in patients infected by multiple DENV serotypes. Number of SFCs after peptide pool stimuli at three concentrations by ELISPOT; different DENV serotypes are specified in the legend; the red-bordered dot indicates the patient at the secondary infection. (**A**) DENV1-4 CD4 MP stimulus; (**B**) DENV1-4 CD8 MP stimulus; (**C**) DENV1 pool; (**D**) DENV2 pool; (**E**) DENV3 pool; (**F**) DENV4 pool. Triangles represent responders to each stimulus. The horizontal bars represent the medians. IFN-γ production was evaluated as the number of SFC detected by ELISPOT after stimulation with three different concentrations of peptide pools. The data were compared using the Wilcoxon U test; the differences were considered significant at *p* ≤ 0.05. Footnotes: CD: cluster definition; MP: MegaPool; DENV: dengue virus; SFC: Spot Forming Cell. The lower section of each graph displays the number of responders and their respective proportions.

Based on the defined criteria, DENV1-4 CD4 MP induced a specific T-cell response in seven out of eight patients (87.5%) at all tested concentrations (*p* > 0.99). However, considering the magnitude of the response, the 1 µg/mL concentration induced a response that nearly reached statistical significance (median 325 SFC/10^6^ cells, IQR 156–475) (*p* = 0.0547) when compared to the 0.1 µg/mL concentration (median 250 SFC/10^6^ cells, IQR 115–344) (Figure 1A). DENV1-4 CD8 MP elicited a T-cell response in four out of eight individuals (50.0%) at the lowest concentration [0.1 µg/mL (median 13 SFC/10^6^ cells, IQR 0–121)], and six responders (75.0%) were observed at both 1 µg/mL (median 53 SFC/10^6^ cells, IQR 10–758) and 5 µg/mL concentrations (median 95 SFC/10^6^ cells, IQR 16–844) (*p* = 0.47). In terms of response magnitude, the DENV1-4 CD8 MP showed a trend of concentration-dependent IFN-γ production (Figure 1B). Importantly, both DENV1-4 CD4 and CD8 MPs, which encompass all four dengue serotypes, elicited specific-T-cell responses in all patients with dengue infection, independent of the infecting serotype (Figure 1A,B).

The serotype-specific DENV peptide pools showed limited T-cell responses in dengue patients, despite the matched serotype. Both DENV1 and DENV2 pools showed a higher number of responders at 5 µg/mL (5/8, 62.5%, for both stimuli) (median 45 SFC/10^6^ cells, IQR 0–49 for DENV1 and median 28 SFC/10^6^ cells, IQR 18–101 for DENV2) compared to lower concentrations [4/8 (50.0%) and 3/8 (37.5%) at both 0.1 µg/mL (median 3 SFC/10^6^ cells, IQR 0–14 for DENV1 and median 5 SFC/10^6^ cells, IQR 0–10 for DENV2) and 1 µg/mL (median 10 SFC/10^6^ cells, IQR 0–35 for DENV1 and median 7 SFC/10^6^ cells, IQR 0–46 for DENV2) for DENV1 and for DENV2 pools, respectively]. Furthermore, only the highest concentration elicited a moderate IFN-γ response (Figure 1C,D). The DENV3 pool failed to induce a specific T-cell response even at the highest concentration (5 µg/mL) (Figure 1E). Finally, the DENV4 pool elicited a measurable T-cell response in four out of eight individuals (50.0%) at the 5 µg/mL concentration (Figure 1F). In contrast, fewer responders were observed at lower concentrations, specifically, one out of eight (12.5%) at 0.1 µg/mL and two out of eight (25.0%) at 1 µg/mL. Notably, the highest concentration was also the only one to induce a minimal IFN-γ response.

The analysis of the response magnitude revealed that DENV1-4 CD4 MP elicited a significantly higher T-cell response compared to the other DENV serotype pools across all concentrations (0.1 µg/mL *p* = 0.002; 1 µg/mL *p* = 0.012; 5 µg/mL *p* = 0.002). In contrast, although the response pattern observed for DENV1-4 CD8 MP resembled that of DENV1-4 CD4 MP, the differences were not statistically significant at any of the concentrations tested.

**Figure 2:**
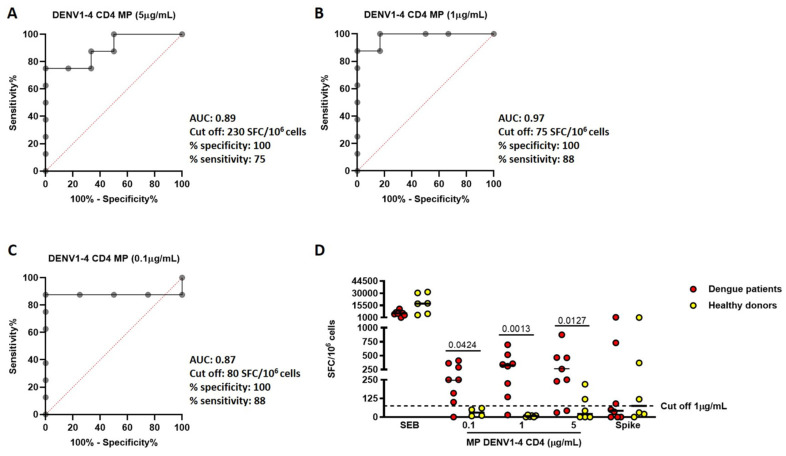
DENV1-4 CD4 MP at 1 µg/mL is the optimal concentration to discriminate dengue-specific T-cell response. (**A**) ROC analysis of DENV1-4 CD4 MP 5 µg/mL; (**B**) ROC analysis of DENV1-4 CD4 MP 1 µg/mL; (**C**) ROC analysis of DENV1-4 CD4 MP 0.1 µg/mL; (**D**) comparison of IFN-γ production in dengue patients (red dots) and healthy donors (yellow dots) using a cut-off of 75 SFC/10^6^ cells. The horizontal bars represent the medians. ROC and analysis of the AUC were applied to define cut-off values for scoring purposes. The data were compared using the Mann–Whitney and Wilcoxon U tests; the differences were considered significant at *p* ≤ 0.05. Footnotes: ROC: Receiver Operating Characteristic; AUC: area under the curve; SEB: Staphylococcal enterotoxin B; CD: cluster definition; MP: MegaPool; DENV: dengue virus; SFC: Spot Forming Cell.

Based on the results, DENV1-4 CD4 and CD8 MPs were selected for further analysis. The accuracy of detecting T-cell-specific responses to DENV1-4 CD4 MP (Figure 2) and DENV1-4 CD8 MP (Figure S2) was evaluated using ROC analysis, with comparisons made between dengue patients and healthy vaccinated donors at all three tested concentrations. For DENV1-4 CD4 MP at 5 µg/mL (AUC = 0.89, *p* = 0.014; Figure 2A), the cut-off 230 SFC/10^6^ cells defined a positive response with a 100% specificity (95% IC: 61–100) and 75% sensitivity (95% IC: 41–96), while for both 1 µg/mL (AUC = 0.97, *p* = 0.003; Figure 2B) and 0.1 µg/mL (AUC = 0.87, *p* = 0.041; Figure 2C), the cut-off 75 SFC/10^6^ cells and 80 SFC/10^6^ cells defined a positive response with a specificity of 100% (95% CI: 53–99 for both) and 88% sensitivity (95% CI: 61–100; 95% CI: 51–100, respectively). Consequently, following the analysis of AUC and accuracy outcomes, the 1 µg/mL concentration was chosen. As a result, the cut-off value was applied to compare individuals diagnosed with dengue disease and healthy donors (Figure 2D). A cut-off value of 75 SFC/10^6^ cells identified seven out of eight dengue patients as responders, while all healthy donors remained below this threshold and were classified as non-responders. This result indicates that stimulating PBMCs with DENV1-4 CD4 MP at 1 µg/mL serves as an effective experimental condition for detecting dengue T-cell-specific responses.

A similar analysis for DENV1-4 CD8 MP indicates that the 5 µg/mL concentration serves as an effective experimental condition for detecting dengue T-cell-specific responses to this antigen (Figure S2).

### 3.3. DENV1-4 CD4 and CD8 MPs Are Suitable Candidates to Evaluate the Dengue-Specific T-Cell Response in Vaccinated Subjects

**Figure 3:**
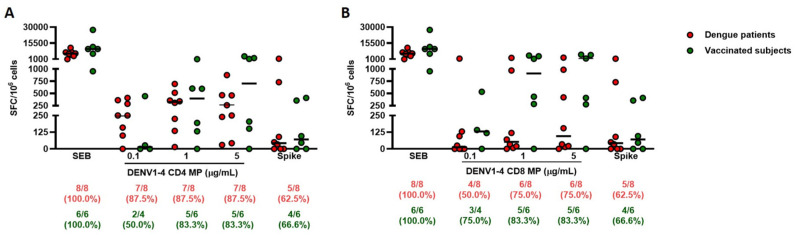
DENV1-4 MPs are effectively able to induce dengue-specific T-cell response in QDENGA-vaccinated subjects. Comparison of SFC measured by ELISPOT following DENV1-4 MPs at three concentrations in dengue patients (red dots) versus QDENGA-vaccinated subjects (green dots). (**A**) DENV1-4 CD4 MP; (**B**) DENV1-4 CD8 MP. The horizontal bars represent the medians. The data were compared using the Mann–Whitney and Wilcoxon U tests; the differences were considered significant at *p* ≤ 0.05. Footnotes: SEB: Staphylococcal enterotoxin B; CD: cluster definition; DENV: dengue virus; MP: MegaPool; SFC: Spot Forming Cell. The lower section of each graph displays the number of responders and their respective proportions.

We also evaluated the dengue-specific T-cell response in cells from healthy donors three months after dengue vaccination with Qdenga^®^ (see Section 2). The T-cell responses induced by the two highest concentrations (1 µg/mL and 5 µg/mL) of DENV1-4 CD4 MP (Figure 3A) were similar in both dengue patients and individuals who received the dengue vaccine [7/8 (87.5%) and 5/6 (83.3%), respectively]. To note, DENV1-4 CD4 MP at the lowest concentration induced a T-cell response only in dengue patients. Conversely, DENV1-4 CD8 MP induced a T-cell response mainly in dengue-vaccinated subjects compared to dengue patients [3/4 (75.0%) at 0.1 µg/mL and 5/6 (83.3%) at both 1 µg/mL and 5 µg/mL for DENV1]. Stimulation with DENV1-4 CD8 MP at 1 µg/mL resulted in a higher T-cell response in vaccinated participants compared to dengue patients. A similar, though not statistically significant, trend was observed with DENV1-4 CD8 MP at 5 µg/mL (Figure 3B). The T-cell responses to SEB and the Spike pool did not significantly differ between the two groups. Using peptides specific to the DENV serotype (Figure S3), no distinct T-cell response was observed in either dengue patients or individuals who received the dengue vaccine (Figure S3), except for DENV2 peptides (Figure S3B).

### 3.4. DENV1-4 MP Stimulation Induces CD4^+^ and CD8^+^ T-Cell-Specific Responses in Dengue-Vaccinated Subjects

**Figure 4:**
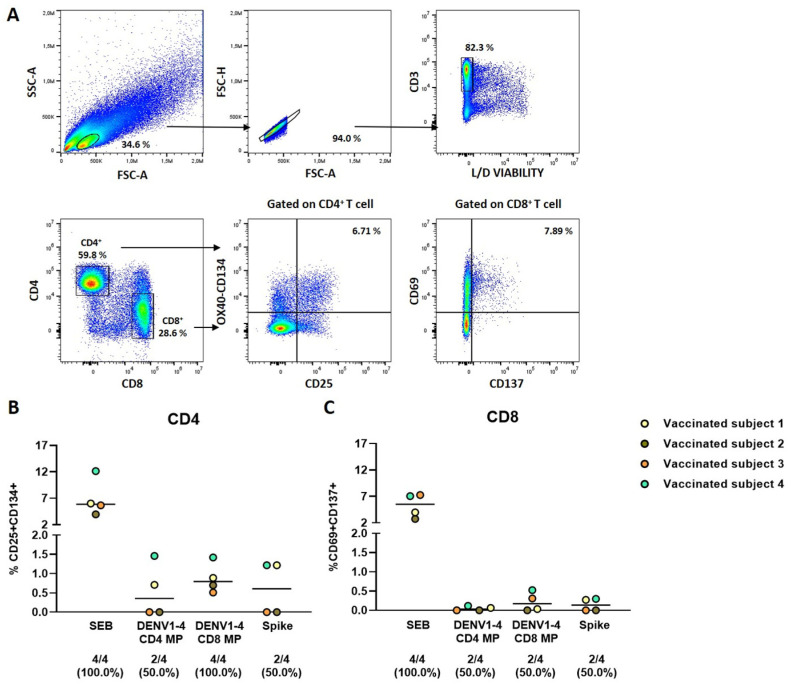
Evaluation of dengue-specific CD4^+^ and CD8^+^ T-cell response in QDENGA-vaccinated subjects following DENV1-4 MP stimulation. (**A**) Flow cytometry gating strategy for CD25^+^CD134^+^CD4^+^ and CD137^+^CD69^+^CD8^+^ T-cells, representative of a vaccinated subject after SEB stimulation; (**B**) antigen-specific response evaluated as CD25^+^CD134^+^CD4^+^ T-cell after 48 h of stimulation; (**C**) antigen-specific response evaluated as CD69^+^CD137^+^CD8^+^ T-cell after 48 h of stimulation. The horizontal bars represent the medians. The data were compared using the Wilcoxon U test; the differences were considered significant at *p* ≤ 0.05. Footnotes: FSC: forward scatter; SSC: side scatter; L/D: Live Dead; SEB: Staphylococcal enterotoxin B; CD: cluster definition; DENV: dengue virus; MP: MegaPool. The lower section of the graph displays the number of responders and their respective proportions.

To evaluate the antigen-specific CD4^+^ and CD8^+^ T-cell response to DENV1-4 MPs, flow cytometry analysis was conducted (gating strategy in Figure 4A). In the dengue patient group, a low number of responders was observed, and only when using DENV1-4 MPs at 5 µg/mL, limiting the characterization of the T-cell response to these MPs. Therefore, we tested DENV1-4 MPs at the 5 µg/mL concentration in four dengue-vaccinated subjects. Antigen-specific responses were assessed by quantifying AIM-positive cells after 48 h of incubation.

Dengue-specific CD4^+^ T-cells, identified by the co-expression of CD25 and CD134, were detected in two out of four (50.0%) vaccinated individuals stimulated with DENV1-4 CD4 MP (percentages are shown in Figure 4B and number of positive cells/10^5^ in Figure S4A). Furthermore, stimulation with DENV1-4 CD8 MP induced dengue-specific CD4^+^ T-cells in 100.0% of vaccinated subjects (Figure 4B). Moreover, as expected, a trend of higher magnitude of response was observed in response to DENV1-4 CD4 MP compared to the DENV1-4 CD8 MP. Dengue-specific CD8^+^ T-cells were identified as AIM-positive, analyzing the expression of CD69 and CD137 markers. Both DENV1-4 MPs induced CD8^+^ T-cell responses in two vaccinated individuals (50.0%), with only one individual responding to the stimulation of both MPs (percentages are shown in Figure 4C and number of positive cells/10^5^ in Figure S4B). Both CD4^+^ and CD8^+^ SEB-specific T-cell responses were observed. Moreover, the magnitude of response of antigen-specific T-cells induced by DENV1-4 MP stimuli in both CD4^+^ and CD8^+^ T-cell populations was similar to that observed with unrelated stimuli used for recall response testing, such as Spike (percentages are shown in Figure 4B,C, and number of positive cells/10^5^ in Figure S4A,B). The corresponding unstimulated controls are provided in Figure S5.

## 4. Discussion

In this study, we tested an experimental tool to evaluate dengue virus (DENV)-specific T-cell response using DENV peptide stimulation. We found that DENV MegaPools (MPs) enabled the detection of robust and reproducible responses mediated by both CD4 and CD8 T-cells. In contrast, selected serotype-specific DENV peptide pools have a lower ability to induce a measurable T-cell response, potentially due to the epitope pool composition. As key components of adaptive immunity, evaluating antigen-specific T-cell responses is crucial for elucidating immunopathology in the context of natural infection and serves as a fundamental aspect of assessing vaccine efficacy [42]. There are several methods to measure antigen-specific T-cell responses, including cytokine-based assays, which are also used in diagnostic tests [e.g., IFN-γ release assays, ELISPOT, and intracellular cytokine staining (ICS)] [43] or AIM assays [44,45]. However, the accuracy of these tests often relies on the quality of the antigen used for measuring the T-cell response [38]. Peptides are recognized as an ideal source of antigen for measuring a T-cell-specific response, as their easy and fast production offers a standardized product with a broad applicability (e.g., whole blood stimulation, AIM, ICS, ELISPOT) [46,47]. Nonetheless, pools of overlapping peptides span the entire sequence of antigens, regardless of which epitopes are more likely recognized in a given human population and of known HLA phenotype [48,49]. In contrast, an MP approach, besides offering comprehensive coverage of a diverse and broad epitope repertoire of selected antigens, also provides exhaustive HLA coverage. Moreover, this approach reduces the number of peptides within the pool, reducing or eliminating toxicity due to the solvent requirement [27]. Collectively, these features render MPs well-suited for comprehensive analyses of T-cell responses to virtually any pathogen, whether in the context of natural infection or vaccination, as demonstrated by multiple studies in the fields of COVID-19 and tuberculosis [40,50,51,52]. In this study, we demonstrate that DENV1-4 MPs, rather than serotype-specific peptide pools, constitute the most effective tool for quantifying the DENV-specific T-cell response, as evidenced by the ELISPOT results. Likely, the inclusion of diverse HLA experimentally defined, used for the MP approach, may have had an impact on the results. Not surprisingly, we detected a DENV-specific T-cell response to both antigen-MPs and serotype-specific DENV pools—regardless of the patient’s DENV serotype—suggesting the induction of serotype-cross-reactive memory T-cells [53].

When comparing DENV-specific T-cell responses, dengue patients show strong responses even at the lowest MP concentrations, unlike vaccinated individuals. This suggests that natural infection leads to higher T-cell avidity due to more effective priming. T-cell functional avidity—measured by the peptide concentration needed for 50% activation—typically increases during immune responses and upon pathogen re-exposure [54,55]. Moreover, most dengue patients (7/8, 87.5%) were at their first dengue infection. Interestingly, the only patient experiencing a reinfection exhibited the lowest response to both DENV1-4 CD4 and CD8 MPs. These findings may suggest a heterologous reinfection in this patient, characterized by suboptimal avidity of T-cells for the epitopes of the novel serotype [56] or inadequate expansion of naïve T-cells against the secondary serotype. Moreover, although the secondary infection, this patient experienced a non-severe dengue. More in-depth characterizations of the immune responses in this patient would be important, both to elucidate the mechanisms underlying T-cell anergy to dengue during reinfection and to clarify their contribution to the non-severe course of disease.

We included a recall antigen as SARS-CoV-2 Spike peptides, since all subjects tested had received at least one dose of a COVID-19 vaccine, to evaluate the specificity of T-cell response in dengue patients and in DENV-vaccinated subjects. In both groups, the T-cell-specific response to Spike is almost always lower compared to the DENV-specific T-cell response, possibly reflecting different times post-exposure or vaccination; moreover, the response to Spike is comparable between DENV-infected and -vaccinated subjects. Thus, while these results suggest the reactivation of SARS-CoV-2 memory T-cells, they also highlight a strong responsiveness of these cells triggered by either natural DENV infection or vaccination, further confirming the effectiveness of DENV1-4 MPs in eliciting this response. Not all tested subjects exhibited a measurable Spike response, and given the limited information available regarding their “COVID-19 status”, it can only be speculated that non-responders to the SARS-CoV-2 antigen either failed to mount an effective T-cell response as previously shown [38], received fewer vaccine doses, experienced fewer breakthrough infections, or were vaccinated earlier than the responders.

The magnitude of the DENV-specific T-cell response, particularly for the MP developed to stimulate CD8 responses, was approximately ten times higher in individuals vaccinated for dengue compared to those naturally infected. These observed differences are independent of the immunocompetence of the individuals assessed, as all participants demonstrated a comparable response to the positive control (SEB). This was true even among those affected by DENV disease, who may experience lymphopenia. These findings are consistent with a recent study that observed CD8^+^ T-cell responses induced by TAK-003 vaccination, targeting all four DENV serotypes [55], where the CD8^+^ T-cell response was found up to three years after vaccination. In this cohort, subjects were assessed three months following vaccination, and the number of individuals included in this study is limited. This study aimed to identify the optimal experimental conditions for assessing T-cell responses elicited by either natural infection or vaccination. The findings presented here will serve as a foundation for future studies aimed at evaluating dengue vaccine efficacy and clarifying the contribution of T-cell responses in these contexts. Further analysis of the CD4^+^ and CD8^+^ T-cell response dynamics throughout the post-vaccination period, particularly after administration of the second vaccine dose, would provide valuable insights.

## 5. Conclusions

In this study, we tested an experimental tool to evaluate the T-cell response specific to the dengue virus (DENV), demonstrating that DENV1-4 MegaPools (MPs) enabled the detection of robust and reproducible antigen-specific responses mediated by both CD4^+^ and CD8^+^ T-cells. These findings provide valuable insights for applying this approach to the study of dengue-specific T-cell immunity during natural infection and for evaluating dengue vaccine efficacy.

## Figures and Tables

**Table 1:** Demographic and clinical characteristics of the study population.

	DENV Patients	Healthy Donors/Vaccinated Subjects	*p*-Value
N (%)	8 (100)	6 (100)	
Median age year (IQR)	42 (25–71)	48 (25–65)	0.83
Female gender N (%)	5 (62.5)	3 (50)	0.64
Origin N (%)			0.19
Italy	6 (75)	6 (100)	
Asia	2 (25)	0 (0)	
Dengue serotypes			
DENV1	2 (25)	-	-
DENV2	3 (37.5)	-	-
DENV3	3 (37.5)	-	-
Serology positivity N (%)			
IgM	6 (75)	0 (0)	-
IgG	6 (75)	0 (0)	-
Median days to symptom onset (IQR)	7 (4–9)	-	-
Dengue severity N (%)			
Severe	0 (0)	-	-
Non-severe	8 (100)	-	-

N = number; IQR = interquartile range; DENV = dengue.

## Data Availability

The raw data generated and/or analyzed within the present study will be available in INMI institutional repository (rawdata.inmi.it), subject to registration. The data can be found by selecting the article of interest from a list of articles ordered by year of publication. No charge for granting access to data is required. In the event of a malfunction of the application, the request can be sent directly by email to biblioteca@inmi.it.

## Supplementary Materials

The following supporting information can be downloaded at: https://www.mdpi.com/article/10.3390/pathogens15010005/s1. Figure S1: SEB and Spike induce IFN-γ-producing T-cells in patients infected with different DENV-serotype. Number of SFC after stimulation by ELISPOT, different DENV-serotypes are indicated in the legend. The horizontal bars represent the medians. Footnotes SEB: Staphylococcal enterotoxin B; DENV: dengue virus; SFC: Spot Forming Cells; Figure S2: Evaluation of the accuracy of DENV CD8 MP in patients diagnosed with dengue infection. (A) ROC analysis of DENV1-4 CD8 MP 5 μg/mL; (B) ROC analysis of DENV1-4 CD8 MP 1 μg/mL; (C) ROC analysis of DENV1-4 CD8 MP 0.1 μg/mL; (D) comparison of IFN-γ production in dengue patients (red dots) and healthy donors (yellow dots) using a cut off of 83 SFC/10^6^ cells. The horizontal bars represent the medians. ROC and analysis of the AUC were applied to define cut-off values for scoring purposes. The data were compared using the Mann–Whitney and Wilcoxon U tests; the differences were considered significant at *p*-values of *p* ≤ 0.05. Footnotes: ROC: Receiver Operating Characteristic; AUC: Area under the curve; SEB: Staphylococcal enterotoxin B; CD: Cluster definition; MP: Megapool; DENV: dengue virus; SFC: Spot Forming Cells; Figure S3: IFN-γ T-cell responses to serotype-specific peptide pools. Comparison of SFC measured by ELISPOT following serotype-specific DENV peptide pools at three concentrations in dengue patients (red dots) versus QDENGA vaccinated subjects (green dots). (A) DENV1 pool; (B) DENV2 pool; (C) DENV3 pool; (D) DENV4 pool. The horizontal bars represent the medians. The data were compared using the Mann–Whitney and Wilcoxon U test; the differences were considered significant at *p*-values of *p* ≤ 0.05. Footnotes: SEB: Staphylococcal enterotoxin B; DENV: dengue virus; SFC: Spot Forming Cells. The lower section of each graph displays the number of responders and their respective proportions; Figure S4: Number of positive cells/10^5^ of dengue-specific CD4^+^ and CD8^+^ T-cell response in QDENGA vaccinated subjects following DENV1-4 MPs stimulation. (A) Antigen-specific response evaluated as CD25^+^CD134^+^CD4^+^ T-cell after 48 h of stimulation; (B) Antigen-specific response evaluated as CD69^+^CD137^+^CD8^+^ T-cell after 48 h of stimulation. The horizontal bars represent the medians. The data were compared using the Wilcoxon U test; the differences were considered significant at *p*-values of *p* ≤ 0.05. Footnotes: SEB: Staphylococcal enterotoxin B; CD: Cluster definition; DENV: dengue virus; MP: Megapool. The lower section of the graph displays the number of responders and their respective proportions; Figure S5: Unstimulated samples for CD4^+^ and CD8^+^ T-cell response in QDENGA vaccinated subjects. (A) Percentage of CD4^+^CD25^+^CD134^+^ and CD8^+^CD69^+^CD137^+^ T-cell after 48 h in unstimulated condition; (B) Number of positive cells/10^5^ cells of CD25^+^CD134^+^CD4^+^ and CD69^+^CD137^+^CD8^+^ T-cell after 48 h in unstimulated condition. The horizontal bars represent the medians. Footnotes: CD: Cluster definition.

## References

[1] Dengue https://www.who.int/news-room/fact-sheets/detail/dengue-and-severe-dengue.

[2] Paz-Bailey G., Adams L.E., Deen J., Anderson K.B., Katzelnick L.C. (2024). Dengue. Lancet Lond. Engl..

[3] Guzman M.G., Harris E. (2015). Dengue. Lancet.

[4] Khan A.M., Heiny A.T., Lee K.X., Srinivasan K.N., Tan T.W., August J.T., Brusic V. (2006). Large-Scale Analysis of Antigenic Diversity of T-Cell Epitopes in Dengue Virus. BMC Bioinform..

[5] Harapan H., Michie A., Sasmono R.T., Imrie A. (2020). Dengue: A Minireview. Viruses.

[6] Halstead S.B. (2007). Dengue. Lancet Lond. Engl..

[7] Khetarpal N., Khanna I. (2016). Dengue Fever: Causes, Complications, and Vaccine Strategies. J. Immunol. Res..

[8] Mongkolsapaya J., Dejnirattisai W., Xu X., Vasanawathana S., Tangthawornchaikul N., Chairunsri A., Sawasdivorn S., Duangchinda T., Dong T., Rowland-Jones S. (2003). Original Antigenic Sin and Apoptosis in the Pathogenesis of Dengue Hemorrhagic Fever. Nat. Med..

[9] Duangchinda T., Dejnirattisai W., Vasanawathana S., Limpitikul W., Tangthawornchaikul N., Malasit P., Mongkolsapaya J., Screaton G. (2010). Immunodominant T-Cell Responses to Dengue Virus NS3 Are Associated with DHF. Proc. Natl. Acad. Sci. USA.

[10] Kalimuddin S., Chia P.Y., Low J.G., Ooi E.E. (2025). Dengue and Severe Dengue. Clin. Microbiol. Rev..

[11] Zompi S., Santich B.H., Beatty P.R., Harris E. (2012). Protection from Secondary Dengue Virus Infection in a Mouse Model Reveals the Role of Serotype Cross-Reactive B and T Cells. J. Immunol..

[12] Weiskopf D., Angelo M.A., de Azeredo E.L., Sidney J., Greenbaum J.A., Fernando A.N., Broadwater A., Kolla R.V., De Silva A.D., de Silva A.M. (2013). Comprehensive Analysis of Dengue Virus-Specific Responses Supports an HLA-Linked Protective Role for CD8+ T Cells. Proc. Natl. Acad. Sci. USA.

[13] Zellweger R.M., Tang W.W., Eddy W.E., King K., Sanchez M.C., Shresta S. (2015). CD8+ T Cells Can Mediate Short-Term Protection against Heterotypic Dengue Virus Reinfection in Mice. J. Virol..

[14] Wang R., Kim B., Mishra H., Kain K.C. (2025). Advancing Dengue Vaccine Development: Challenges, Innovations, and the Path toward Global Protection. Pediatr. Investig..

[15] Aynekulu Mersha D.G., van der Sterren I., van Leeuwen L.P.M., Langerak T., Hakim M.S., Martina B., van Lelyveld S.F.L., van Gorp E.C.M. (2024). The Role of Antibody-Dependent Enhancement in Dengue Vaccination. Trop. Dis. Travel Med. Vaccines.

[16] Kuczera D., Assolini J.P., Tomiotto-Pellissier F., Pavanelli W.R., Silveira G.F. (2018). Highlights for Dengue Immunopathogenesis: Antibody-Dependent Enhancement, Cytokine Storm, and Beyond. J. Interferon Cytokine Res. Off. J. Int. Soc. Interferon Cytokine Res..

[17] de Barros Cardoso C.R., Cerqueira-Silva T., Barral-Netto M., Boaventura V.S. (2025). Dengue Dilemma: Navigating Cross-Reactivity and Immune Challenges. Current Topics in Microbiology and Immunology.

[18] St John A.L., Rathore A.P.S. (2019). Adaptive Immune Responses to Primary and Secondary Dengue Virus Infections. Nat. Rev. Immunol..

[19] Mathew A., Rothman A.L. (2008). Understanding the Contribution of Cellular Immunity to Dengue Disease Pathogenesis. Immunol. Rev..

[20] Singha S., Nath N., Sarma V., Barman K., Sharma G.C., Saikia L., Baruah S. (2024). Identification of Immunodominant Epitopes of Dengue Virus 2 Envelope and NS1 Proteins: Evaluating the Diagnostic Potential of a Synthetic Peptide. Mol. Diagn. Ther..

[21] Khan A.M., Miotto O., Nascimento E.J.M., Srinivasan K.N., Heiny A.T., Zhang G.L., Marques E.T., Tan T.W., Brusic V., Salmon J. (2008). Conservation and Variability of Dengue Virus Proteins: Implications for Vaccine Design. PLoS Negl. Trop. Dis..

[22] Tian Y., Grifoni A., Sette A., Weiskopf D. (2019). Human T Cell Response to Dengue Virus Infection. Front. Immunol..

[23] Verma M., Bhatnagar S., Kumari K., Mittal N., Sukhralia S., Gopirajan At S., Dhanaraj P.S., Lal R. (2019). Highly Conserved Epitopes of DENV Structural and Non-Structural Proteins: Candidates for Universal Vaccine Targets. Gene.

[24] Gonçalves Pereira M.H., Figueiredo M.M., Queiroz C.P., Magalhães T.V.B., Mafra A., Diniz L.M.O., da Costa Ú.L., Gollob K.J., Antonelli L.R.d.V., Santiago H.d.C. (2020). T-Cells Producing Multiple Combinations of IFNγ, TNF and IL10 Are Associated with Mild Forms of Dengue Infection. Immunology.

[25] Sanchez-Vargas L.A., Anderson K.B., Srikiatkhachorn A., Currier J.R., Friberg H., Endy T.P., Fernandez S., Mathew A., Rothman A.L. (2021). Longitudinal Analysis of Dengue Virus–Specific Memory T Cell Responses and Their Association With Clinical Outcome in Subsequent DENV Infection. Front. Immunol..

[26] Goletti D., Petrone L., Manissero D., Bertoletti A., Rao S., Ndunda N., Sette A., Nikolayevskyy V. (2021). The Potential Clinical Utility of Measuring Severe Acute Respiratory Syndrome Coronavirus 2-Specific T-Cell Responses. Clin. Microbiol. Infect. Off. Publ. Eur. Soc. Clin. Microbiol. Infect. Dis..

[27] da Silva Antunes R., Weiskopf D., Sidney J., Rubiro P., Peters B., Lindestam Arlehamn C.S., Grifoni A., Sette A. (2023). The MegaPool Approach to Characterize Adaptive CD4+ and CD8+ T Cell Responses. Curr. Protoc..

[28] Weiskopf D., Cerpas C., Angelo M.A., Bangs D.J., Sidney J., Paul S., Peters B., Sanches F.P., Silvera C.G.T., Costa P.R. (2015). Human CD8+ T-Cell Responses Against the 4 Dengue Virus Serotypes Are Associated With Distinct Patterns of Protein Targets. J. Infect. Dis..

[29] Grifoni A., Angelo M.A., Lopez B., O’Rourke P.H., Sidney J., Cerpas C., Balmaseda A., Silveira C.G.T., Maestri A., Costa P.R. (2017). Global Assessment of Dengue Virus-Specific CD4+ T Cell Responses in Dengue-Endemic Areas. Front. Immunol..

[30] Huang C.-H., Tsai Y.-T., Wang S.-F., Wang W.-H., Chen Y.-H. (2021). Dengue Vaccine: An Update. Expert Rev. Anti-Infect. Ther..

[31] Rivera L., Biswal S., Sáez-Llorens X., Reynales H., López-Medina E., Borja-Tabora C., Bravo L., Sirivichayakul C., Kosalaraksa P., Martinez Vargas L. (2022). Three-Year Efficacy and Safety of Takeda’s Dengue Vaccine Candidate (TAK-003). Clin. Infect. Dis..

[32] Huang C.Y.-H., Kinney R.M., Livengood J.A., Bolling B., Arguello J.J., Luy B.E., Silengo S.J., Boroughs K.L., Stovall J.L., Kalanidhi A.P. (2013). Genetic and Phenotypic Characterization of Manufacturing Seeds for a Tetravalent Dengue Vaccine (DENVax). PLoS Negl. Trop. Dis..

[33] Weiskopf D., Angelo M.A., Bangs D.J., Sidney J., Paul S., Peters B., de Silva A.D., Lindow J.C., Diehl S.A., Whitehead S. (2015). The Human CD8+ T Cell Responses Induced by a Live Attenuated Tetravalent Dengue Vaccine Are Directed against Highly Conserved Epitopes. J. Virol..

[34] Tian Y., Babor M., Lane J., Seumois G., Liang S., Goonawardhana N.D.S., De Silva A.D., Phillips E.J., Mallal S.A., da Silva Antunes R. (2019). Dengue-Specific CD8+ T Cell Subsets Display Specialized Transcriptomic and TCR Profiles. J. Clin. Investig..

[35] Gálvez R.I., Martínez-Pérez A., Escarrega E.A., Singh T., Zambrana J.V., Balmaseda Á., Harris E., Weiskopf D. (2025). Frequency of Dengue Virus-Specific T Cells Is Related to Infection Outcome in Endemic Settings. JCI Insight.

[36] Jaiswal S., Pazoles P., Woda M., Shultz L.D., Greiner D.L., Brehm M.A., Mathew A. (2012). Enhanced Humoral and HLA-A2-Restricted Dengue Virus-Specific T-Cell Responses in Humanized BLT NSG Mice. Immunology.

[37] Ambuel Y., Young G., Brewoo J.N., Paykel J., Weisgrau K.L., Rakasz E.G., Haller A.A., Royals M., Huang C.Y.-H., Capuano S. (2014). A Rapid Immunization Strategy with a Live-Attenuated Tetravalent Dengue Vaccine Elicits Protective Neutralizing Antibody Responses in Non-Human Primates. Front. Immunol..

[38] Aiello A., Coppola A., Vanini V., Petrone L., Cuzzi G., Salmi A., Altera A.M.G., Tortorella C., Gualano G., Gasperini C. (2022). Accuracy of QuantiFERON SARS-CoV-2 Research Use Only Assay and Characterization of the CD4+ and CD8+ T Cell-SARS-CoV-2 Response: Comparison with a Homemade Interferon-γ Release Assay. Int. J. Infect. Dis. IJID Off. Publ. Int. Soc. Infect. Dis..

[39] Bettelli F., Vallerini D., Lagreca I., Barozzi P., Riva G., Nasillo V., Paolini A., D’Amico R., Forghieri F., Morselli M. (2024). Identification and Validation of Diagnostic Cut-Offs of the ELISpot Assay for the Diagnosis of Invasive Aspergillosis in High-Risk Patients. PLoS ONE.

[40] Farroni C., Altera A.M.G., Salmi A., Vanini V., Cuzzi G., Lindestam Arlehamn C.S., Sette A., Delogu G., Palucci I., Sbarra S. (2024). Specific Immune Response to M. Tuberculosis and Ability to in Vitro Control Mycobacterial Replication Are Not Impaired in Subjects with Immune-Mediated Inflammatory Disease and Tuberculosis Infection. Front. Immunol..

[41] Escalante P., Peikert T., Van Keulen V.P., Erskine C.L., Bornhorst C.L., Andrist B.R., McCoy K., Pease L.R., Abraham R.S., Knutson K.L. (2015). Combinatorial Immunoprofiling in Latent Tuberculosis Infection. Toward Better Risk Stratification. Am. J. Respir. Crit. Care Med..

[42] Wilder-Smith A. (2020). Dengue Vaccine Development by the Year 2020: Challenges and Prospects. Curr. Opin. Virol..

[43] Tian C., Chen Y., Liu Y., Wang S., Li Y., Wang G., Xia J., Zhao X.-A., Huang R., Lu S. (2018). Use of ELISpot Assay to Study HBs-Specific B Cell Responses in Vaccinated and HBV Infected Humans. Emerg. Microbes Infect..

[44] Dan J.M., Havenar-Daughton C., Silvestri G., Sette A., Crotty S. (2016). Response to Comment on “A Cytokine-Independent Approach To Identify Antigen-Specific Human Germinal Center T Follicular Helper Cells and Rare Antigen-Specific CD4+ T Cells in Blood”. J. Immunol..

[45] Poloni C., Schonhofer C., Ivison S., Levings M.K., Steiner T.S., Cook L. (2023). T-Cell Activation-Induced Marker Assays in Health and Disease. Immunol. Cell Biol..

[46] Petrone L., Vanini V., Amicosante M., Corpolongo A., Gomez Morales M.A., Ludovisi A., Ippolito G., Pozio E., Teggi A., Goletti D. (2017). A T-Cell Diagnostic Test for Cystic Echinococcosis Based on Antigen B Peptides. Parasite Immunol..

[47] Sbarra S., Vola A., Tamarozzi F., Najafi-Fard S., Ludovisi A., Teggi A., Nicastri E., Albarello F., Brunetti E., Goletti D. (2025). Stage-Specific Immune Responses to AgB T-Peptides in Patients with Cystic Echinococcosis. Infect. Dis. Rep..

[48] Maecker H.T., Dunn H.S., Suni M.A., Khatamzas E., Pitcher C.J., Bunde T., Persaud N., Trigona W., Fu T.M., Sinclair E. (2001). Use of Overlapping Peptide Mixtures as Antigens for Cytokine Flow Cytometry. J. Immunol. Methods.

[49] Li Pira G., Ivaldi F., Moretti P., Manca F. (2010). High Throughput T Epitope Mapping and Vaccine Development. J. Biomed. Biotechnol..

[50] Petrone L., Petruccioli E., Vanini V., Cuzzi G., Najafi Fard S., Alonzi T., Castilletti C., Palmieri F., Gualano G., Vittozzi P. (2021). A Whole Blood Test to Measure SARS-CoV-2-Specific Response in COVID-19 Patients. Clin. Microbiol. Infect. Off. Publ. Eur. Soc. Clin. Microbiol. Infect. Dis..

[51] Tarke A., Sidney J., Kidd C.K., Dan J.M., Ramirez S.I., Yu E.D., Mateus J., da Silva Antunes R., Moore E., Rubiro P. (2021). Comprehensive Analysis of T Cell Immunodominance and Immunoprevalence of SARS-CoV-2 Epitopes in COVID-19 Cases. Cell Rep. Med..

[52] Petrone L., Peruzzu D., Altera A.M.G., Salmi A., Vanini V., Cuzzi G., Coppola A., Mellini V., Gualano G., Palmieri F. (2024). Therapy Modulates the Response to T Cell Epitopes over the Spectrum of Tuberculosis Infection. J. Infect..

[53] Friberg H., Bashyam H., Toyosaki-Maeda T., Potts J.A., Greenough T., Kalayanarooj S., Gibbons R.V., Nisalak A., Srikiatkhachorn A., Green S. (2011). Cross-Reactivity and Expansion of Dengue-Specific T Cells during Acute Primary and Secondary Infections in Humans. Sci. Rep..

[54] Viganò S., Utzschneider D.T., Perreau M., Pantaleo G., Zehn D., Harari A. (2012). Functional Avidity: A Measure to Predict the Efficacy of Effector T Cells?. Clin. Dev. Immunol..

[55] Mandaric S., Friberg H., Saez-Llorens X., Borja-Tabora C., Biswal S., Escudero I., Faccin A., Gottardo R., Brose M., Roubinis N. (2024). Long Term T Cell Response and Safety of a Tetravalent Dengue Vaccine in Healthy Children. npj Vaccines.

[56] Rothman A.L. (2011). Immunity to Dengue Virus: A Tale of Original Antigenic Sin and Tropical Cytokine Storms. Nat. Rev. Immunol..

