# Supplementary material for: Peptide MegaPools Approach to Evaluate the Dengue-Specific CD4 and CD8 T-Cell Response
Source: Pathogens. 2025 Dec 20;15(1):5. doi: 10.3390/pathogens15010005 (PMC12845116; doi:10.3390/pathogens15010005)
Supplement: Supplementary file 1 [file pathogens-15-00005-s001.zip › pathogens-3983039-supplementary.pdf]

Supplementary figure S1

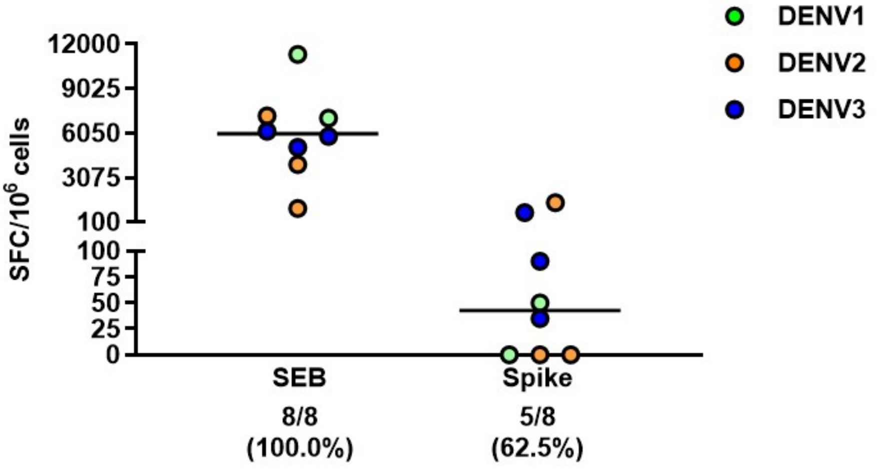

Figure S1. SEB and Spike induce IFN-γ-producing T-cells in patients infected with different DENV-serotype. Number of SFC after stimulation by ELISPOT, different DENV-serotypes are indicated in the legend. The horizontal bars represent the medians. **Footnotes** SEB: Staphylococcal enterotoxin B; DENV: dengue virus; SFC: Spot Forming Cells.

Supplementary figure S2

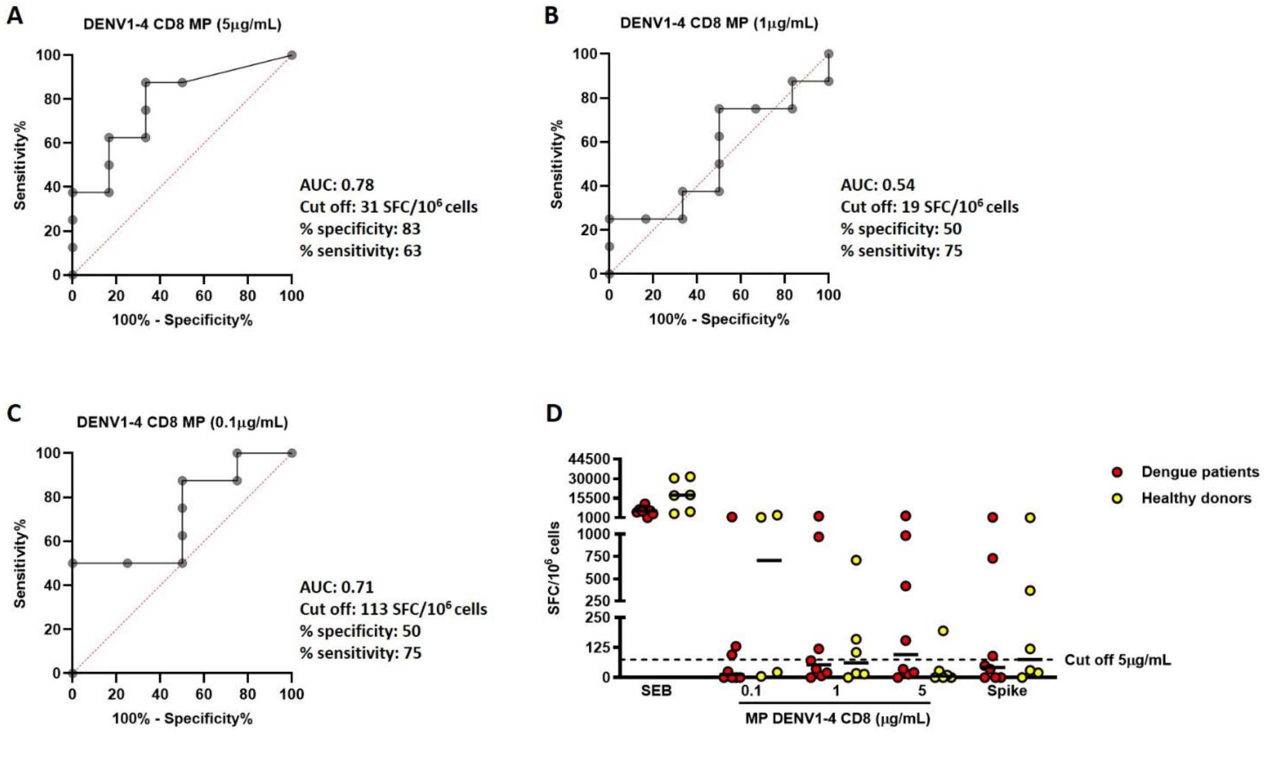

Figure S2. Evaluation of the accuracy of DENV CD8 MP in patients diagnosed with dengue infection. (**A**) ROC analysis of DENV1-4 CD8 MP 5 μg/mL; (**B**) ROC analysis of DENV1-4 CD8 MP 1 μg/mL; (**C**) ROC analysis of DENV1-4 CD8 MP 0.1 μg/mL; (**D**) comparison of IFN-γ production in dengue patients (red dots) and healthy donors (yellow dots) using a cut off of 83 SFC/10^6 cells. The horizontal bars represent the medians. ROC and analysis of the AUC were applied to define cut-off values for scoring purposes. The data were compared using the Mann–Whitney and Wilcoxon U tests; the differences were considered significant at p-values of p≤0.05. **Footnotes:** ROC: Receiver Operating Characteristic; AUC: Area under the curve; SEB: Staphylococcal enterotoxin B; CD: Cluster definition; MP: Megapool; DENV: dengue virus; SFC: Spot Forming Cells.

Supplementary figure S3

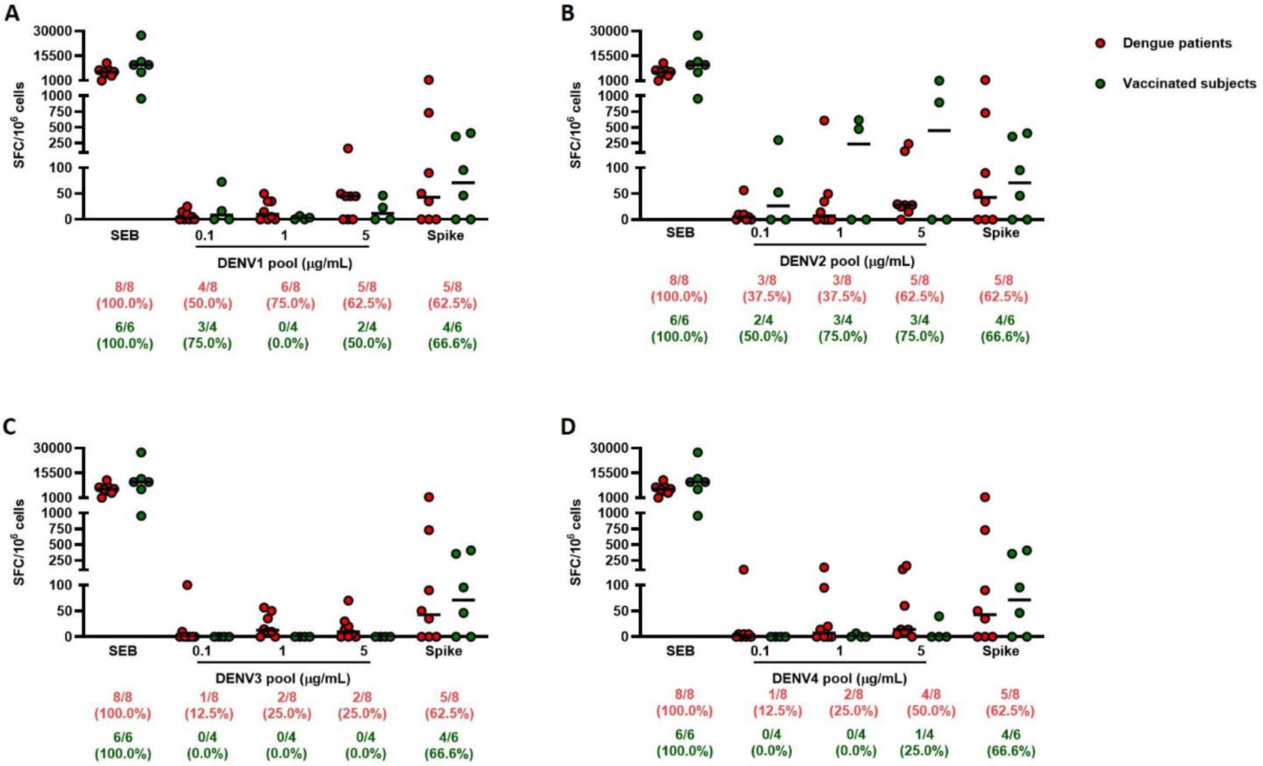

Figure S3. IFN-γ T-cell responses to serotype-specific peptide pools. Comparison of SFC measured by ELISPOT following serotype-specific DENV peptide pools at three concentrations in dengue patients (red dots) versus QDENGA vaccinated subjects (green dots). (**A**) DENV1 pool; (**B**) DENV2 pool; (**C**) DENV3 pool; (**D**) DENV4 pool. The horizontal bars represent the medians. The data were compared using the Mann–Whitney and Wilcoxon U test; the differences were considered significant at p-values of $p \leq 0.05$. **Footnotes:** SEB: Staphylococcal enterotoxin B; DENV: dengue virus; SFC: Spot Forming Cells. The lower section of each graph displays the number of responders and their respective proportions.

Supplementary figure S4

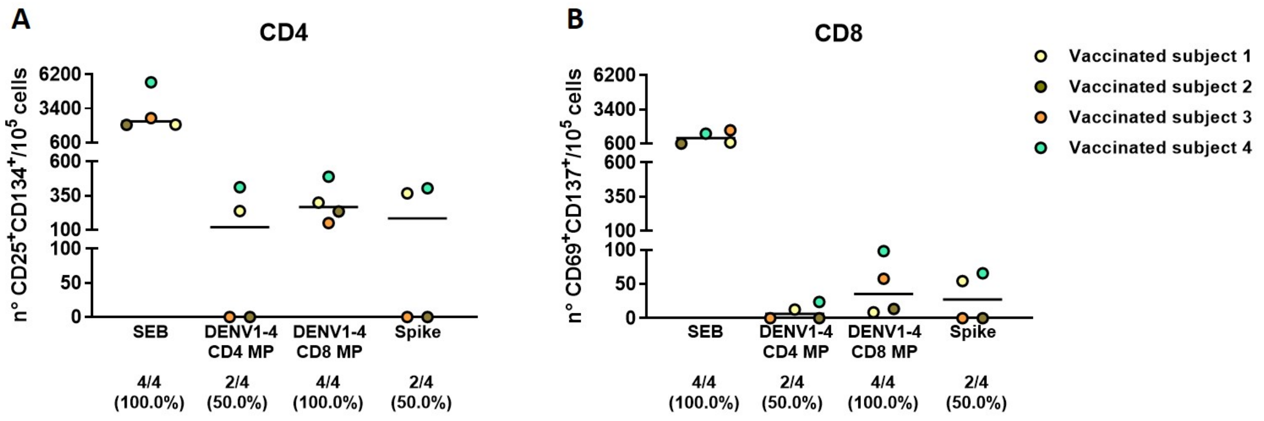

Figure S4. Number of positive cells/10^5 of dengue-specific $CD4^+$ and $CD8^+$ T-cell response in QDENGA vaccinated subjects following DENV1-4 MPs stimulation. (**A**) Antigen-specific response evaluated as $CD25^+CD134^+CD4^+$ T-cell after 48h of stimulation; (**B**) Antigen-specific response evaluated as $CD69^+CD137^+CD8^+$ T-cell after 48h of stimulation. The horizontal bars represent the medians. The data were compared using the Wilcoxon U test; the differences were considered significant at p-values of $p \leq 0.05$. **Footnotes:** SEB: Staphylococcal enterotoxin B; CD: Cluster definition; DENV: dengue virus; MP: Megapool. The lower section of the graph displays the number of responders and their respective proportions.

Supplementary figure S5

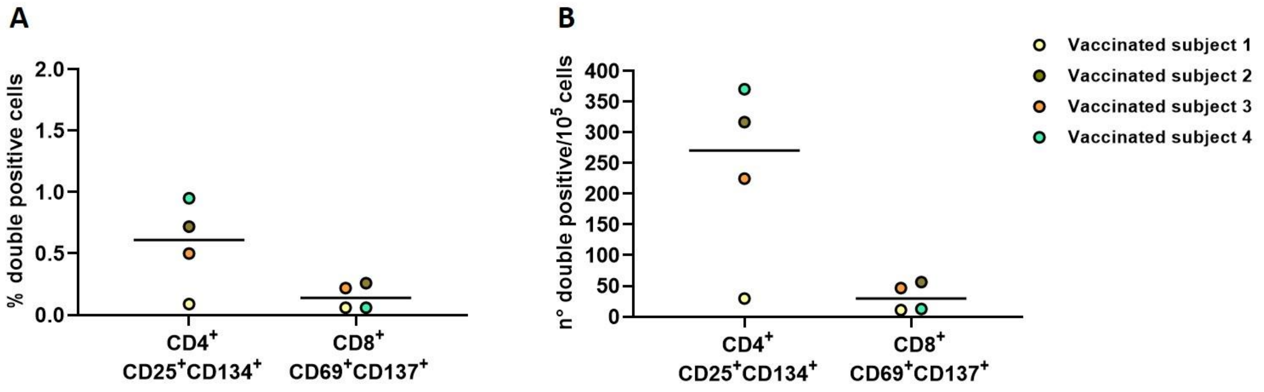

Figure S5. Unstimulated samples for $CD4^+$ and $CD8^+$ T-cell response in QDENGA vaccinated subjects. (**A**) Percentage of $CD4^+CD25^+CD134^+$ and $CD8^+CD69^+CD137^+$ T-cell after 48h in unstimulated condition; (**B**) Number of positive cells/ 10^5 cells of $CD25^+CD134^+CD4^+$ and $CD69^+CD137^+CD8^+$ T-cell after 48h in unstimulated condition. The horizontal bars represent the medians. **Footnotes:** CD: Cluster definition.
